# Sex-Related Anti-Nociceptive Activity of a Flavonoid-Based Formulated Extract from Citrus Peels (Gold Lotion): New Insights into a Rat Model

**DOI:** 10.3390/foods14162877

**Published:** 2025-08-19

**Authors:** Alessandro Di Cerbo, Mario Nicotra, Michiko Suzawa, Tommaso Iannitti, Julio Cesar Morales-Medina

**Affiliations:** 1 School of Biosciences and Veterinary Medicine, University of Camerino, 62024 Matelica, Italy; alessandro.dicerbo@unicam.it (A.D.C.); mario.nicotra@unicam.it (M.N.); 2 Miyauchi Citrus Research Center, Shigoka-Machi, Takasaki 370-3501, Gunma, Japan; michiko@goldcpe.co.jp; 3 Section of Experimental Medicine, Department of Medical Sciences, University of Ferrara, Via Fossato di Mortara 70, 44121 Ferrara, Italy; 4 Centro de Investigación en Reproducción Animal, Cinvestav-Universidad Autónoma de Tlaxcala, AP 62, Tlaxcala 90000, Mexico

**Keywords:** carrageenan, Complete Freund’s Adjuvant, flavonoid, inflammation, mechanical allodynia

## Abstract

Inflammatory pain is a worldwide health problem, affecting primarily women. Nonetheless, most research conducted in this field has involved male subjects, and only 10% of the results have been obtained using females. Gold Lotion (GL) is a formulated product made from the peels of six citrus fruits that have been proven, via experimental data, to possess several biological properties, such as anti-inflammatory, anticancer, and antibacterial activities. This research aims to investigate the response to GL administration in two models of peripheral inflammation to assess potential sex differences. Carrageenan was used to induce mechanical allodynia and maximal edema within hours, serving as a model of acute inflammation, while Complete Freund’s Adjuvant (CFA) was used to obtain similar results within days, resulting in a model of chronic inflammation. Acute GL administration reduced mechanical allodynia in both models and both sexes, with no anti-inflammatory effects observed. In conclusion, this study demonstrates the potential of GL to alleviate inflammatory pain in both males and females, thereby broadening its therapeutic applications.

## 1. Introduction

Inflammation is the immune system’s reaction to harmful chemical, physical, or biological stimuli and can be acute or chronic [1,2]. Acute inflammation is the organism’s early response to minimize impending injury or infection, lasting from minutes to a few days. On the other hand, chronic inflammation occurs when the acute response fails to eliminate the cause of the inflammation and may persist for weeks to years [1,2,3]. One of the consequences of inflammatory reactions is the increased sensitivity of perception and emotional response to noxious stimuli, leading to the development of inflammatory pain [4,5]. The primary role of acute inflammation-associated pain is to prevent reinjury, thereby facilitating a quick recovery. In contrast, chronic inflammatory pain is characterized by clinical signs such as hyperalgesia, allodynia, and spontaneous pain [4,6].

Inflammation-related pain is a complex phenomenon related to several etiological factors, including injuries, infections, autoimmune diseases, and chronic inflammatory illnesses [7,8]. Different studies have shown that the severity and prevalence of pain are higher in women than in men [9,10,11]. In total, 20% of the adult global population suffers from chronic pain, and about half of chronic pain conditions are commonly diagnosed in women, while a smaller percentage is observed in men [10]. For instance, in the UK, 38% of women experienced chronic pain, whereas the percentage among men was 30% [12]. The prevalence was also related to age, lifestyle, and body weight, increasing from 18% in people aged 16–34 years to 53% among the population aged 75 years or more and decreasing in people with a healthy weight and who report completing at least 30 min of moderate or vigorous physical activity per week.

Currently, nonsteroidal anti-inflammatory drugs are mainly employed in the treatment of inflammatory pain, although their prolonged use is associated with gastrointestinal and cardiovascular side effects, such as dyspepsia, gastric mucosal erosions, ulcerations, subepithelial hemorrhage, thrombosis, congestive heart failure, and palpitations [13,14,15]. Nevertheless, natural products (e.g., citrus fruits, roots, or nuts) from traditional Chinese medicine have been shown to hold great anti-inflammatory potential due to the presence of metabolites, e.g., flavonoids, terpenes, *hydroquinones*, steroids, glycosides, alkaloids, carotenoids, and phenolic compounds, with fewer or weaker side effects than drugs [16,17,18].

Citrus fruits represent one of the most widely cultivated crops globally, with an estimated production of over 158 million tons in 2020 and approximately 15 million tons of waste [19,20]. Approximately 30 million tons of citrus fruits are processed into juice annually, resulting in citrus peel that corresponds to almost 50% of the wet fruit mass as a by-product [21]. Citrus peels are also commonly used in Kampo, a traditional Japanese herbal medicine, where they are known as “Chinpi” or “Kippi” and employed for their abundant bioactive compound content [22]. In particular, citrus peels were successfully used in producing Gold Lotion (GL), a commercially available beverage made from six different citrus fruit peels (navel oranges, *Citrus hassaku*, *Citrus limon*, *Citrus natsudaidai*, *Citrus miyauchi*, and *Citrus Satsuma*) with high flavonoid [Nobiletin (50.8 mg/mL), Sinesetin (21.3 mg/mL), Tangeretin (10.6 mg/mL), Naringin (253.6 mg/mL), and Hesperidin (104.7 mg/mL)] and polymethoxylflavone [3,5,6,7,8,3,4-Hepta-methoxyflavone (19.2 mg/mL), 3,5,6,7,3,4-Hexamethoxyflavone (3.1 mg/mL), and 5,6,7,4-Tetramethoxyflavone (1.1 mg/mL)] content and endowed with anti-inflammatory, pain-modulating, antioxidant, and antitumor properties [23,24,25,26,27,28,29]. Oral administration of GL (200 μL) reduced the expression of COX-2 and iNOS in azoxymethane-induced colonic tumor tissues in mice [25]. In addition, GL decreased the expression of pro-inflammatory cytokines TNF-α, interleukin (IL)-6, and IL-12 in lipopolysaccharide (LPS)-stimulated bone marrow-derived macrophage cultures from mice [29]. These previously reported anti-inflammatory actions of GL may, at least in part, underlie the anti-nociceptive effects of GL *in vivo*.

**Figure 1:**
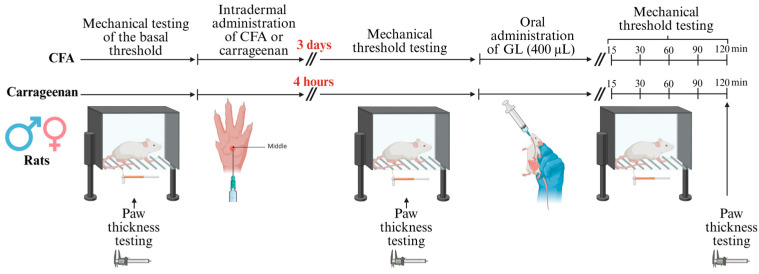
Schematic diagram of procedures. CFA, complete Freund’s Adjuvant; GL, gold lotion.

Despite the widely acknowledged difference between males and females in sensitivity and response to inflammation-related pain and pharmacological treatments, most studies focus on male animal models. In this sense, it would be of great interest to ascertain the fundamental differences in the pain-modulating and anti-inflammatory responses in male and female animal models to any treatment. This research aimed to investigate the aforementioned differences in acute and chronic inflammation of male and female murine models treated with the natural anti-inflammatory product GL (Figure 1).

## 2. Materials and Methods

### 2.1. Animals and Housing

Male (n = 20) and female (n = 21) Wistar rats weighting 230–250 g were obtained from the Centro de Investigacion en Reproduccion Animal (Cinvestav, Tlaxcala, Mexico) and housed at constant temperature and humidity (20 °C and 40% UR), with a 12 h light/dark cycle and food and water *ad libitum*. Three to four rats were housed per standard cage. All procedures complied with the National Institutes of Health Guide for the Care and Use of Laboratory Animals, the technical guidelines for animals in the laboratory issued by SAGARPA Mexico (NOM-062 ZOO-1999), and ARRIVE guidelines [30].

### 2.2. Gold Lotion

GL was kindly provided by the Miyauchi Citrus Research Center (Gunma, Japan) and stored at 4 °C before use. It was administered orally, via gavage, at a dose of 400 µL, according to our previous study [31].

### 2.3. Mechanical Threshold Testing

Animals were acclimated individually in plexiglass boxes containing a stainless steel grid for 1 h before being tested for baseline mechanical allodynia using Von Frey monofilaments (Stoelting Inc., IL, USA) [32]. All tests started at 9:00 a.m. The mid-plantar region of the left hind paw was stimulated using an incremental series composed of eight monofilaments of logarithmic stiffness to determine the 50% withdrawal threshold through the modified version of the Dixon up–down method [33]. Briefly, a Von Frey filament (number 4.31, force = 2 g) was applied perpendicularly to the plantar skin, causing slight bending. Then, rapid paw withdrawal within 6 s was considered a positive response, so the procedure was repeated using a larger filament. Conversely, if the response was negative, no withdrawal happened, and a larger filament was used. Paw thickness was determined using a caliper.

### 2.4. Complete Freund’s Adjuvant (CFA) Model

On day 0, the animals belonging to the CFA group underwent the administration of CFA supplemented with 1 mg/mL *Mycobacterium tuberculosis* (Sigma, St. Louis, MO, USA; 50 μL) to the ventral mid-plantar region of their left hind paw. Mechanical responses and paw edema were measured 3 days post-injection on the left hind paw. On day 3, rats received either GL (400 µL) or a vehicle (saline) orally by gavage. Then, the mechanical threshold was assessed at 15, 30, 60, 90, and 120 min post-treatment using the Von Frey filaments as previously described [7,31,32,^34^,]. Paw edema was measured prior to GL administration on day 3 and the 120 min post-treatment.

### 2.5. Carrageenan Model

Carrageenan (Sigma, C1867-5G) was dissolved in saline, heated to 37 °C, and vortexed to prepare a 3% carrageenan solution, which was then stored at 4 °C until use. Similarly, as in the CFA group on day 0, 50 μL of the 3% carrageenan solution or saline was injected intradermally into the left hind paw of all subjects in the carrageenan group. Mechanical responses were assessed at 15, 30, 60, 90, and 120 min after the GL or vehicle injection (400 µL). Paw edema was measured at the peak of inflammation and 120 min post-treatment with GL [7,31,32,^34^].

### 2.6. Statistical Analysis

Data were analyzed using GraphPad Prism 9.5.1 (GraphPad Software Inc., San Diego, CA, USA). Mechanical threshold and paw edema data were analyzed using a two-way analysis of variance (ANOVA) followed by Dunnett’s post hoc test for multiple comparisons. The maximum change in mechanical allodynia (E_max_%) [35] and Area Under the Curve (AUC) [36] data were analyzed using a multiple unpaired *t*-test. A *p* < 0.05 was considered significant.

## 3. Results

### 3.1. GL Improves Carrageenan- and CFA-Induced Mechanical Sensitivity in Males and Females

**Figure 2:**
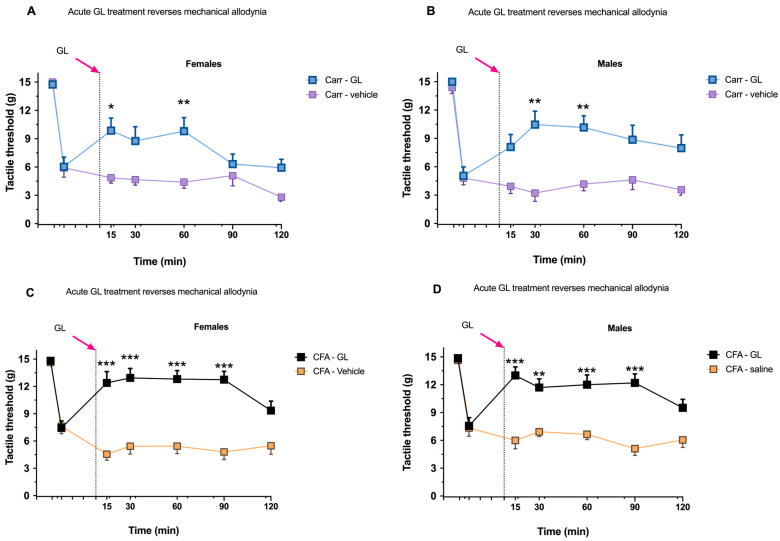
The effects of GL administration or saline were observed 4 h after carrageenan administration or 3 days after CFA injection. (**A**,**B**) The mechanical response threshold at baseline and 4 h post-carrageenan administration, followed by measurements at 15, 30, 60, 90, and 120 min post-GL or saline administration in females and males, respectively. (**C**,**D**) The mechanical response threshold at baseline and Day 3 after the administration of CFA and post-GL or saline administration at 15, 30, 60, 90, and 120 min in females and males, respectively. * *p* < 0.05; ** *p* < 0.01; *** *p* < 0.001. Data are presented as mean ± SEM.

The administration of carrageenan and CFA decreased the tactile threshold compared to the baseline in both males and females, thus indicating an increase in mechanical allodynia. However, some differences between the sexes were observed after the administration of GL. Concerning the carrageenan group, female subjects showed a significant improvement in mechanical allodynia at 15 and 60 min post-GL compared to the carrageenan/vehicle group (9.85 ± 1.36, *p* < 0.05, and 9.79 ± 1.48, respectively, *p* < 0.01; Figure 2A). Also, males showed a similar improvement at 30 and 60 min post-GL administration (10.46 ± 1.47 and 10.15 ± 1.36, respectively, *p <* 0.01; Figure 2B) compared to the control group.

Regarding the CFA group, an improvement in mechanical allodynia was also observed. Specifically, in GL-treated females, the tactile threshold increased at 15, 30, 60, and 90 min post-GL administration (12.39 ± 1.23, 12.94 ± 1.04, 12.81 ± 0.92, and 12.73 ± 0.92, respectively, *p* < 0.001; Figure 2C). Similarly, in GL-treated male mice, the same increase was observed at 15, 30, 60, and 90 min after the administration of GL (12.99 ± 0.92 *p* < 0.001, 11.70 ± 0.92 *p* < 0.01, 11.99 ± 1.05 *p* < 0.001, and 12.19 ± 0.97 *p* < 0.001, respectively; Figure 2D).

**Figure 3:**
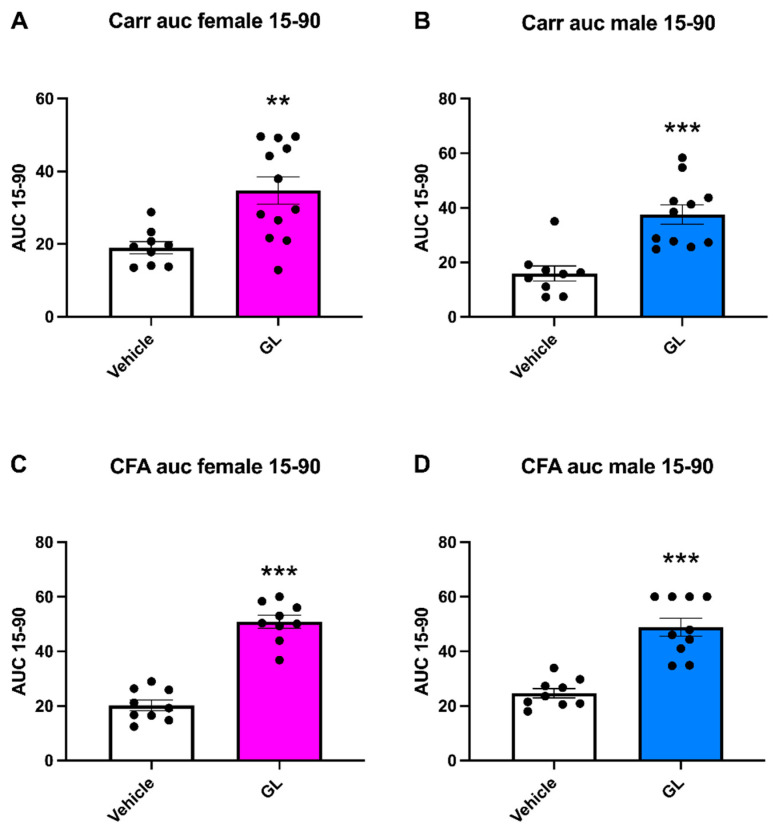
Graphical representation of the AUC data of carrageenan- and CFA-induced mechanical hypersensitivity. (**A**,**B**) The AUC of data from Figure 2A,B for the period between 15 and 90 min post-GL or vehicle (PBS) administration in females and male rats. (**C**,**D**) The AUC of data from Figure 2C,D for 15 and 90 min post-GL or vehicle (PBS) administration in female and male rats. ** *p* < 0.01, *** *p* < 0.001.

The mechanical allodynia results were also confirmed by the AUC analysis between 15 and 90 min after GL administration (Figure 3A–D). Notably, in the carrageenan model, a significant increase in the AUC of the GL group in comparison to the vehicle was reported in males (37.58 ± 3.55 cm^2^ and 15.94 ± 2.76 cm^2^, respectively; *p* < 0.001) and females (34.72 ± 3.75 cm^2^ and 19.1 ± 1.66 cm^2^, respectively; *p* < 0.01). Regarding the CFA model, both sexes exhibited a significant increase. In particular, in males, the AUC increased from 24.69 ± 1.70 cm^2^ in the vehicle group to 48.89 ± 3.30 cm^2^ in the GL group (*p* < 0.001), while in females, it increased from 20.22 ± 1.92 cm^2^ in the vehicle group to 50.89 ± 2.41 cm^2^ in the GL group (*p* < 0.001).

### 3.2. Efficacy of GL in CFA and Carrageenan Models

**Figure 4:**
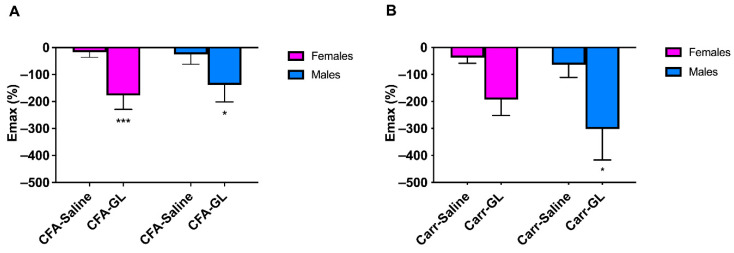
Graphical representation of the E_max_% (maximum effect observed presented as a percentage) and the mechanical threshold in the ipsilateral paw after Gold Lotion treatment, calculated from (**A**) 3 days and (**B**) 4 h after the administration of CFA or carrageenan. * *p* < 0.05, *** *p* < 0.001.

A significant increase in the E_max_% was observed in males and females of the CFA model, and in males of the carrageenan model. In particular, in the CFA model, the E_max_ significantly decreased from −17.40 ± 18.59% to −177.4 ± 51.55% (*p* < 0.001) in females and from −25.12 ± 12.31% to −138.0 ± 63.42% (*p* < 0.05) in males (Figure 4A). Regarding the carrageenan model, the E_max_ decreased from −64.15 ± 46.73% to −302.0 ± 114.7% in males, while no significant difference was observed in females (Figure 4B).

### 3.3. Gold Lotion Does Not Influence Paw Edema in Both Carrageenan- and CFA-Induced Mechanical Hypersensitivity

**Figure 5:**
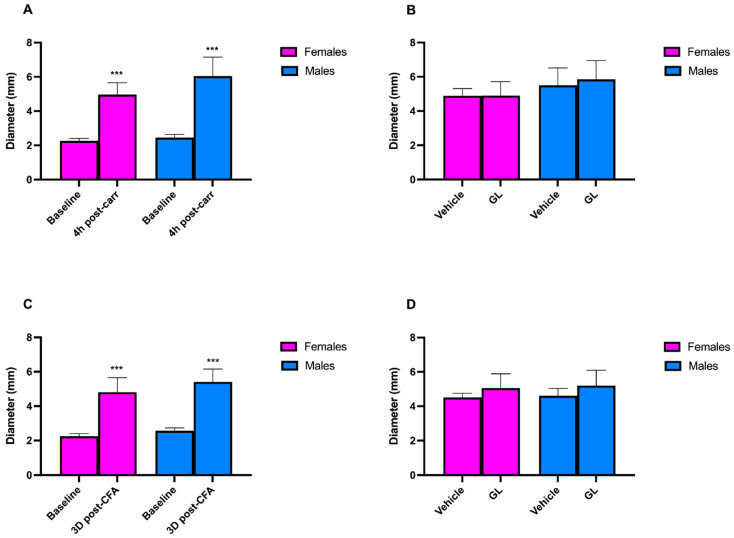
Graphical representation of the diameter of paw edema. (**A**,**C**) The mean diameter of the left hind paw at baseline (BL), 4 h, and 3 days after carrageenan or CFA administration. (**B**,**D**) The mean diameter of the left hind paw after administering vehicle (PBS) or GL in carrageenan- and CFA-treated mice. *** *p* < 0.001.

The administration of carrageenan and CFA induced a significant increase in paw edema in males and females after 4 h and 3 days, respectively. Concerning the carrageenan model, the diameter increased from 2.26 ± 0.03 mm to 4.97 ± 0.15 mm in females, while in males, from 2.45 ± 0.04 mm to 6.04 ± 0.24 mm (*p* < 0.001; Figure 5A). On the other hand, 3 days after the administration of CFA, the paw edema increased from 2.24 ± 0.03 mm to 4.80 ± 0.20 mm in females and from 2.56 ± 0.03 mm to 5.41 ± 0.17 mm in males (*p* < 0.001; Figure 5C). The administration of GL did not result in a reduction in paw edema in males and females (Figure 5B,D).

## 4. Discussion

Pain is a global public health priority [37]. Yearly, one in five adults suffers from chronic or recurrent pain, and one in ten is diagnosed with chronic pain [38]. The latter impacts mostly women; nevertheless, most pain studies are conducted on male models [39]. One of the causes of pain can be inflammation, as some mediators involved in the inflammatory process, specifically prostaglandins, pro-inflammatory cytokines, and chemokines, can activate nociceptors and lead to the detection of noxious stimuli [40]. Recently, functional foods have garnered the attention of both consumers and researchers for their ability to meet nutritional requirements while providing health benefits [41]. Moreover, from a circular economy perspective, agricultural waste is gaining popularity as a source of nutraceuticals for use in disease management. In this context, the administration of GL produced analgesic-like effects in both male and female rats across two models of inflammatory pain, with observed differences in pharmacodynamic responses.

Different studies have demonstrated the beneficial effects of GL. For instance, it was shown that GL can downregulate the protein levels of inflammation-related proteins (iNOS and COX-2) [25]. Another study conducted in 2014 suggested GL as a potential therapeutic agent in the treatment of chronic inflammation and autoimmune diseases due to its ability to impair the function of dendritic cells [29]. These molecules play a central role in inflammation, pain, and the immune responses associated with them. In our previous study, we assessed anxiety- and depression-related behaviors, nociception, and inflammation following repeated administration of GL in male rats. While GL effectively reversed inflamogen-induced mechanical allodynia with limited inflammatory effects, it did not influence anxiety- or depression-related behaviors. In the present study, we extended our investigation to include both male and female rats, evaluating their responses to two well-established pro-inflammatory agents. Interestingly, the acute administration of GL did not modulate the paw edema.

Among GL constituents, the most abundant are flavonoids, such as Nobiletin, Sinesetin, Tangeretin, Naringin, Hesperidin, 3,5,6,7,8,3′, 4′-Heptamethoxyflavone, 3,5,6,7,3′, 4′-Hexamethoxyflavone, and 5,6,7,4′-Tetramethoxyflavone [29]. Each molecule contains specific biological activities; for example, nobiletin has been proven to interfere with the production of PGE_2_ and induce a reduction in the expression of the mRNAs of some pro-inflammatory cytokines (IL-1α, IL-1β, TNF-α, and IL-1β) in mouse macrophages [26], while tangeretin has been associated with a potent antioxidant and anti-inflammatory effect in microglia [27]. Furthermore, nobiletin and 3,5,6,7,8,3′,4′-heptamethoxyflavone have shown the capability to contrast TPA-induced skin inflammation and suppress the expression of COX-2 induced by exposure to UVB [28]. The synergistic effects of these molecules contribute to the analgesic-related properties observed in GL.

It is widely acknowledged that women are more sensitive to pain and exhibit a reduction in pain inhibition and an enhancement in pain facilitation, likely due to an interaction between various hormonal, anatomical, and genetic factors [42,43,44]. Moreover, females respond differently from males to pharmacological treatments due to factors such as their lower body weight and higher body fat percentage, slower glomerular filtration rate, and the influence of sex hormones that can affect the pharmacokinetics and pharmacodynamics of drugs [45]. For example, it has been shown that ibuprofen is more effective in controlling pain in men, while ketorolac is more effective in women. Studies concerning gender differences in opioids, specifically morphine, have produced contrasting results [46,47,48]. Nevertheless, due to concerns that the hormonal cycle in females may modify behavioral outcomes [45,49], most studies still use male animal models. Mice and rats are the most widely used animal models in pain studies, while larger mammals, such as dogs and pigs, are rarely used in preclinical pain studies [50]. Different studies have demonstrated the existence of sex-related differences in response to pain and analgesia, which can be attributed to genetic factors, anatomical characteristics, and the role of gonadal hormones [51]. An anatomic difference that can influence the response to pain is the representation of fat; in fact, the greater percentage of body fat in adult male mice compared to females can affect the distribution of some analgesics, thereby interfering with their potency or duration of action. Regarding genetic factors, microglia in the spinal cord are not required for mechanical pain in mice [52]. Concerning gonadal hormones, estradiol and testosterone have been shown to modulate sensitivity to pain and analgesia [53]. For example, it was shown that estrogens can interact with the opioid system and induce the internalization of the μ-opioid receptor in the medial preoptic nucleus and the medial amygdala [54]. Since there are numerous flavonoids in GL, it is possible to postulate that various flavonoids contribute to the analgesic-related effects using different mechanisms, and therefore, we could observe analgesic-related effects in both male and female rats.

## 5. Conclusions

Our findings demonstrate that the acute administration of GL elicits significant anti-nociceptive effects in rats, independent of sex and the type of inflammatory agent used, as assessed from a behavioral pharmacology perspective. These results highlight the potential of GL as a broadly effective analgesic agent in inflammatory pain models and support its further investigation for sex-inclusive pain management strategies.

Further studies will need to assess changes in markers of pain and inflammation following GL administration. This will shed light on why GL displayed anti-nociceptive effects but not anti-inflammatory effects in the present study.

## Data Availability

The original contributions presented in this study are included in the article. Further inquiries can be directed to the corresponding author.

## Abbreviations

The following abbreviations are used in this manuscript:

GL	Gold Lotion
CFA	Complete Freund’s Adjuvant
AUC	Area Under the Curve
